# Supplementary material for: Cell-Surface Programmed Death Ligand-1 Expression Identifies a Sub-Population of Distal Epithelial Cells Enriched in Idiopathic Pulmonary Fibrosis
Source: Cells. 2022 May 10;11(10):1593. doi: 10.3390/cells11101593 (PMC9139571; doi:10.3390/cells11101593)
Supplement: Supplementary file 1 [file cells-11-01593-s001.zip › cells-1653371-supplementary.pdf]

cells

MDPI

Article

Cell-Surface Programmed Death Ligand-1 Expression Identifies a Sub-Population of Distal Epithelial Cells Enriched in Idiopathic Pulmonary Fibrosis

Negah Ahmadvand [1,†], Gianni Carraro [2,†], Matthew R. Jones [1], Irina Shalashova [1], Afshin Noori [1], Jochen Wilhelm [3], Nelli Baal [4], Farhad Khosravi [5], Chengshui Chen [6], Jin-San Zhang [6], Clemens Ruppert [1,7], Andreas Guenther [1,7,8], Roxana M. Wasnick [1,‡,*] and Saverio Bellusci [6,9,‡,*]

[1] Cardio-Pulmonary Institute, Department of Pulmonary and Critical Care Medicine and Infectious Diseases, Universities of Giessen and Marburg Lung Center (UGMLC), Member of the German Center for Lung Research (DZL), Justus-Liebig University Giessen, 35392 Giessen, Germany; negah.ahmadvand@innere.med.uni-giessen.de (N.A.); matthew.jones@innere.med.uni-giessen.de (M.R.J.); irn.bry@yandex.ru (I.S.); afshin.noori@med.uni-giessen.de (A.N.); clemens.ruppert@innere.med.uni-giessen.de (C.R.); andreas.guenther@innere.med.uni-giessen.de (A.G.)

[2] Lung and Regenerative Medicine Institutes, Cedars-Sinai Medical Center, Department of Medicine, Los Angeles, CA 90048, USA; gianni.carraro@csmc.edu

[3] Institute for Lung Health (ILH), Department of Internal Medicine, Justus-Liebig University Giessen, 35392 Giessen, Germany; jochen.wilhelm@patho.med.uni-giessen.de

[4] Department of Clinical Immunology and Transfusion Medicine, 35392 Giessen, Germany; nelli.baal@immunologie.med.uni-giessen.de

[5] Department of Physiology, Justus-Liebig University Giessen, 35392 Giessen, Germany; farhad.khosravi@physiologie.med.uni-giessen.de

[6] The Quzhou Affiliated Hospital of Wenzhou Medical University, Quzhou People's Hospital, Quzhou 324000, China; chenchengshui@wmu.edu.cn (C.C.); zhang_jinsan@163.com (J.-S.Z.)

[7] European IPF Registry/UGLMC Giessen Biobank, 35392 Giessen, Germany

[8] Lung Clinic Waldhof-Elgershausen, 35753 Greifenstein, Germany

[9] Laboratory of Extracellular Lung Matrix Remodelling, Department of Internal Medicine, Universities of Giessen and Marburg Lung Center (UGMLC), Member of the German Center for Lung Research (DZL), Justus-Liebig University Giessen, 35392 Giessen, Germany

* Correspondence: roxana.wasnick@cellergon.de (R.M.W.); saverio.bellusci@innere.med.uni-giessen.de (S.B.)

† These authors contributed equally to this work.

‡ These authors contributed equally to this work.

Supplementary Materials: The following are available online at https://www.mdpi.com/article/10.3390/cells11101593/s1.

Table S1: Antibodies used for flow cytometry staining

Table S2: qPCR primers

Figure S1: Expression of CD274 mRNA in donor and IPF lung

Figure S2: Expression of FGFR2B and ETV5 in donor and IPF-derived CD274^{pos} cells

Figure S3: Cell surface expression of CD274 in donor and IPF human lung

Figure S4: Transcriptomic analysis of donor and IPF-derived AT2 and CD274^{pos} cell populations

Supplementary Tables

Table S1. Antibodies used for flow cytometry staining

Primary antibody target	Company	Cat no	FACS (per 10*6 cells)
anti human proSP-B	Millipore	AB3430	1_200
anti human EpCAM APC-Cy7	Biolegend	324222	0.5_100
anti human CD45 Pe-Cy7	Biolegend	304016	2.5_100
anti human CD31 Pe-Cy7	Biolegend	303120	0.5_100
anti human HTII-280	TerraceBiotech	TB-27AHT2-280	1_500
anti human CD274	Biolegend	374514	1_100
Donkey anti-rabbit Alexa-Fluor 488	ThermoFisherScientific	A21206	1_500
anti mouse EpCAM Pe-Cy7	Biolegend	118216	0.25_100
anti mouse CD45 APC-Cy7	Biolegend	103114	0.25_100
anti mouse CD31 APC-Cy7	Biolegend	102418	0.25_100
anti mouse CD274 unconjugated	Thermo-Fischer	PA5-20343	1_100
Goat anti rabbit antibody Alexa flour 488 (Invitrogen,1:500)	ThermoFisherScientific	A11008	1_500

Table S2. qPCR primers

Gene	Forward primer (5'->3')	Reverse primer (5'->3')
**(Human)**	CCTAAGATGAGCGCAAGTTGAA	CCACAGGACTAGAACACCTGCTAA
GAPDH	ACCCAGAAGACTGTGGATGG	GTGTCGCTGTTGAAGTCAGAG
SCGB1A1	CCCTCCTCCACCATGAAACTC	AGGGTTTCGATGACACGCTG
ETV5	AGGGGCAGAAAACCACCAAA	GTCCCGTTTTGCGGGTACTA
FGFR2b	GATAAATAGTTCCAATGCAGAAGTGCT	TGCCCTATATAATTGGAGACCTTACA
SFTPC	CACTGAAGCGGGGTCATCCA	TGCAAAAGCTGCAAAAGACCC
CD274	TGGCATTTGCTGAACGCATTT	AGTGCAGCCAGGTCTAATTGT
**Gene (Mouse)**	**Forward primer (5'->3')**	**Reverse primer (5'->3')**
Hprt	CCTAAGATGAGCGCAAGTTGAA	CCACAGGACTAGAACACCTGCTAA
Sftpc	GGTCCTGATGGAGAGTCCAC	GATGAGAAGGCGTTTGAGG
Cd274	AAGTCAATGCCCCATACCGC	TTCTGGATAACCCTCGGCCT
Scgb1a1	GCCTCCAACCTCTACCATGA	TCAGGGATGCCACATAACCA

Supplementary figures

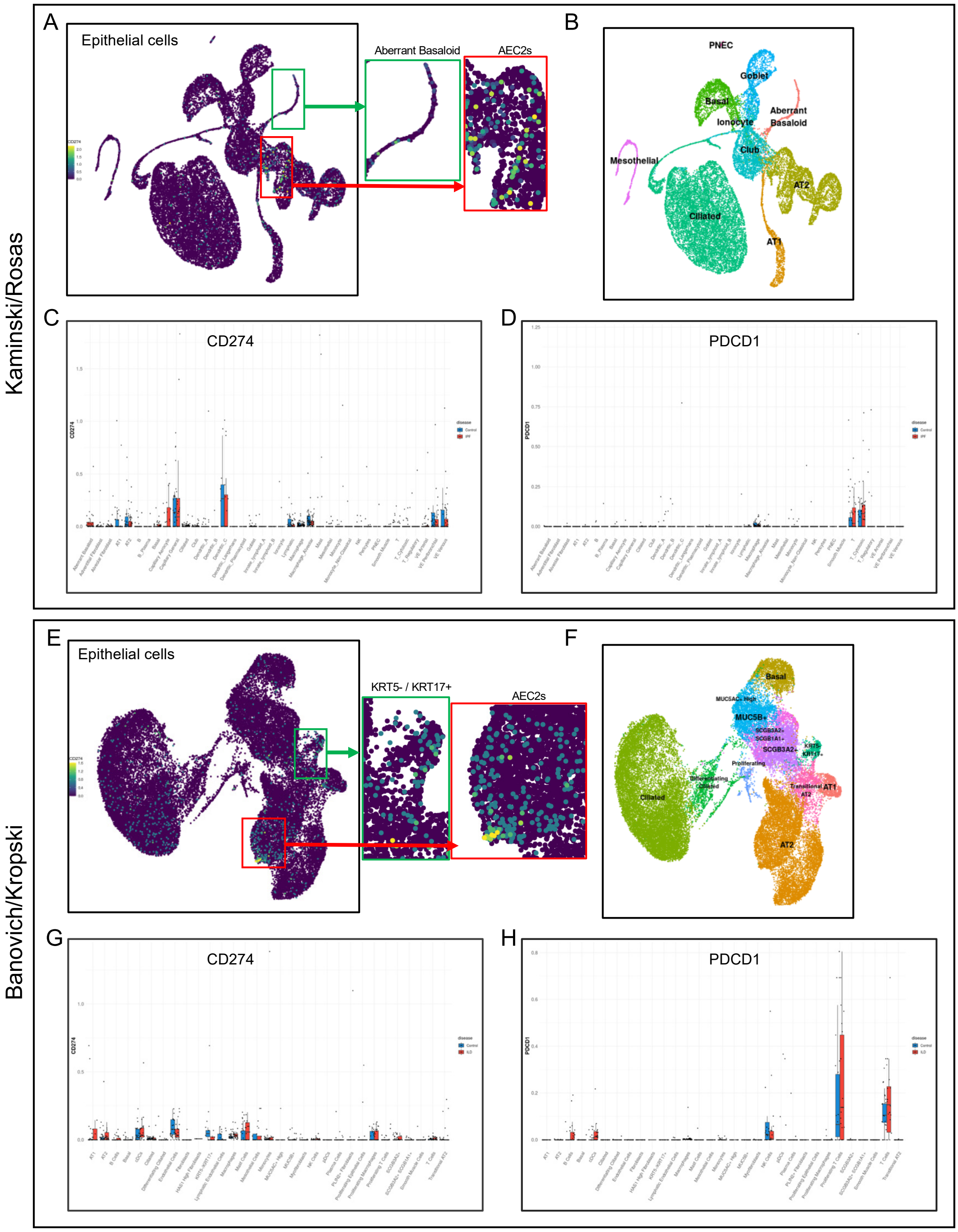

Figure S1. **Expression of** ***CD274*** **mRNA in donor and IPF lungs.** Alveolar epithelium expression of *CD274* mRNA in the Kaminski/Rosas (A) and Banovich/Kropski (E) data sets shown as UMAPs. (B, F) UMAP representation of different epithelial cell type clusters in the Kaminski/Rosas (B), and Banovich/Kropski (F) data sets. (C, G) Overview of the *CD274* mRNA expression levels in donor and IPF throughout all lung cell types. (D, H) Expression of *PDCD1* (*PD1*) in the immune cell compartment of donor and IPF lungs in the Kaminski/Rosas (D), and Banovich/Kropski (H) data sets. Data retrieved from www.ipfcellatlas.com.

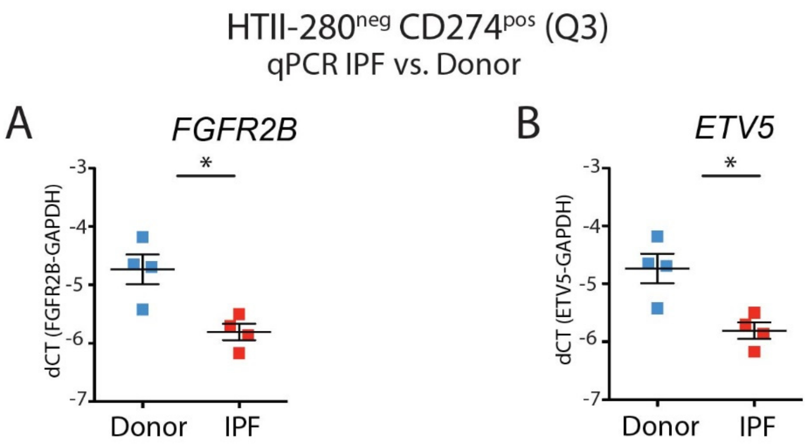

Figure S2. Expression of *FGFR2B* and *ETV5* in donor and IPF-derived CD274pos cells. (A) qPCR analysis of *FGFR2B* in HTII-280neg CD274pos cells (Q3) subpopulations in donors (n=4) and IPF (n=4). (B) qPCR analysis of *ETV5* in HTII-280neg CD274pos cells (Q3) subpopulations in donors (n=4) and IPF (n=4). Data are represented as means ± SEM. $^{*}p < 0.05$, $^{**} p < 0.01$, $^{***}p < 0.001$, $^{****}p < 0.0001$.

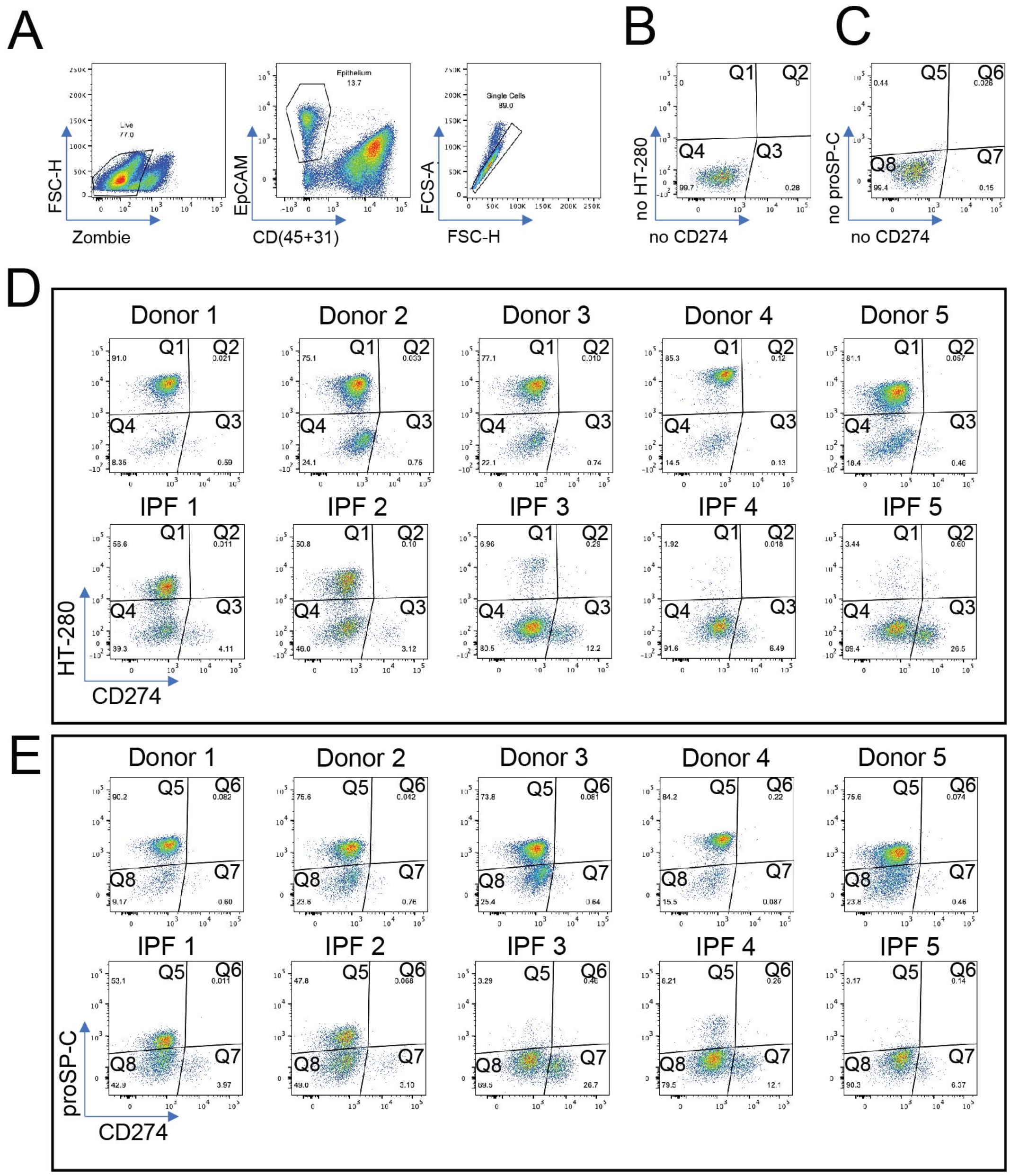

Figure S3. Cell surface expression of CD274 in donor and IPF human lung. Five donor and five end-stage IPF lung samples were dissociated into single cell suspension and analyzed by flow-cytometry. (A) Representative flow cytometry panels showing the gating strategy of the epithelial cells analyzed in Figure 2. First, live cells were identified as Zombie^{neg} cells (left panel), then epithelial cells were identified as CD45^{neg} CD31^{neg} EpCAM^{pos} (middle panel) and doublets were excluded based on FSC-A vs FSC-H analysis (right panel). (B, C) Controls where HTII-280 and CD274 (left panel) and proSP-C and HTII-280 were omitted were used to define the gating for the flow cytometry analysis in Figure 2. (D) Individual panels for each of the patients in the HTII-280 CD274 analysis. (E) Individual panels for each of the patients in the proSP-C CD274 analysis.

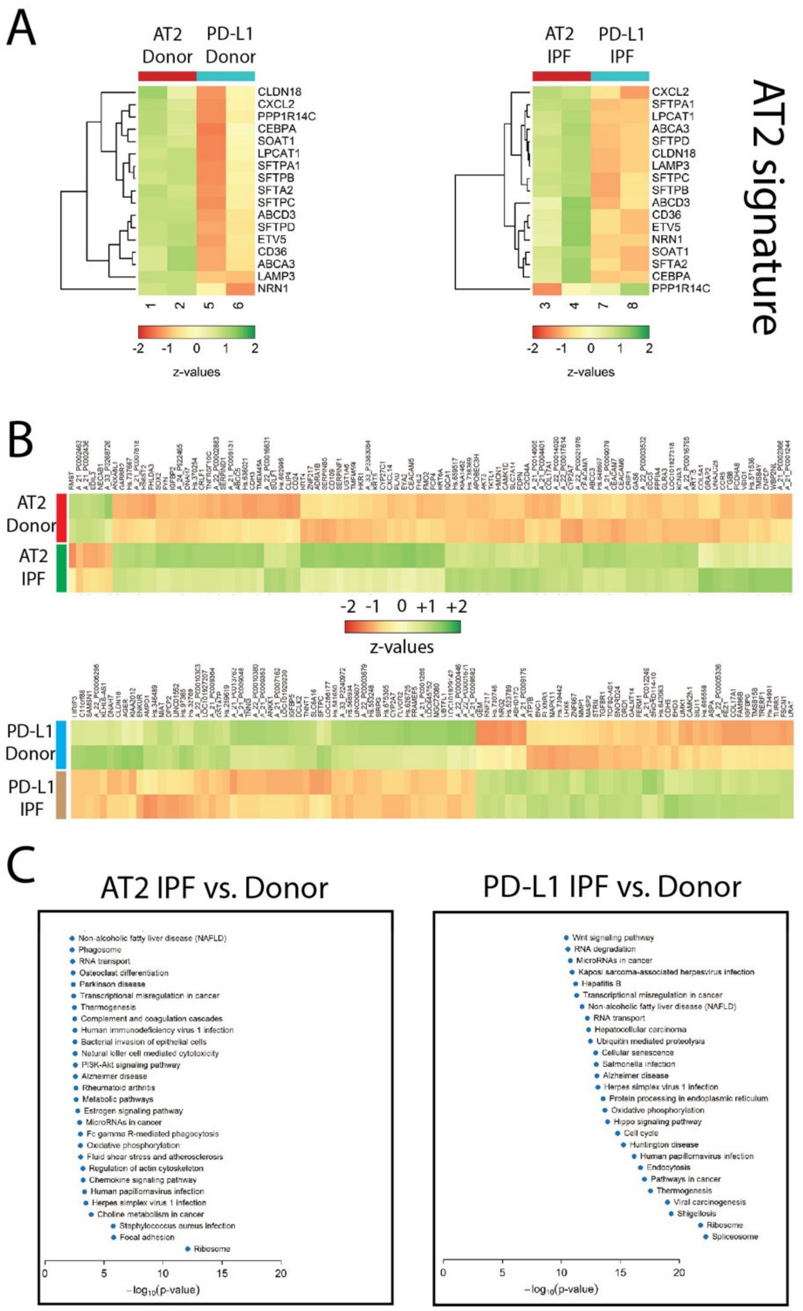

Figure S4. Transcriptomic analysis of donor and IPF-derived AT2 and CD274^{pos} (PD-L1^{pos}) cell populations. (A) Heatmaps of AT2 cell signature genes based on the microarray data of isolated AT2 and PD-L1^{pos} cells in both donor (n=2) and IPF (n=2) lungs. (B) Heatmap of the top 100 differentially regulated genes in AT2 cells (according to the p-value) in donor vs. IPF, and heatmap of the top 100 differentially regulated genes in PD-L1^{pos} cells (according to the p-value) in donor vs. IPF. (C) Corresponding KEGG pathway analysis showing the top regulated pathways in AT2 and PD-L1^{pos} cells in IPF vs. donor, according to significance ($-\log_{10}$(p-value)).
